# Grants for Nurse-Training Schools

**Published:** 1921-09-24

**Authors:** 


### THE MATRONS' AND SISTERS' DEPARTMENT.

### GRANTS FOR NURSE-TRAINING SCHOOLS.

THE General Nursing Council, by the issue of its Syllabus of training, has done much to bring into notice the weighty educational work which devolves on the training-schools for nurses maintained by the hospitals. Long before the official declaration of the course of instruction nurses must undergo in order to fit them for their duties, the hospitals had been vying one with another to improve the opportunities afforded to their nurses. It is now a very long time since the course was so enlarged as to comprise a great number of subjects not strictly needed by the nurse in order to perform her duties in the hospital.

Many nurses can remember the days when very ignorant women, possessed of a mere smattering of nursing lore, sufficed to carry out the mechanical part of the work. It was not in former days thought necessary, or even desirable, to teach more than the most ordinary nomenclature of medical and surgical theory to probationers who did not aspire to become sisters. The education of the probationers was bounded by the actual needs of the hospital service, and there can be no doubt that the enlargement of the nurse's field of knowledge has brought in its train a very considerable addition to the expenses of the hospital. Nurses are now trained with an eye to their subsequent career. And in fitting them for the public service hospitals have a very strong claim to the public assistance granted to almost every other educational establishment not run for private gain.

The educational work of the hospitals begins at a point where it has been plainly left undone by the educational authorities. The difficulties of the training-school would be enormously reduced if such a standard of education had been attained by the ordinary candidate as to fit her to attend lectures without someone being obliged to explain them all over again afterwards and reduce them as it were, to pap; if she were able to take notes of what she heard and express herself comprehensibly; if she could answer questions clearly and had learned the art of observation. The matron gets her candidates imperfectly educated, and it is the educational authorities of the country who ought to make good the deficiencies, not the overburdened hospitals supported by private charity.

But beyond the elements of education needed by the nurse if she is to be of use after leaving the wards, we come to other subjects not strictly needed in hospital, but indispensable to the nurse in her after-work. Why, it may surely be asked, must the hospitals be at the expense of teaching their nurses cookery, for instance, when during their term of training they will never require to practise it? The thorough grounding in medical and surgical nursing now rightly deemed indispensable for the probationer is not, strictly speaking, indispensable to her while working in a subordinate position in the wards. So far from the hospital needing a large number of nurses trained in theatre work, it is one of the acutest problems of the matron to ensure all her nurses a sufficient training in this department. The same might be said of gynæcology and other special subjects. The hospital aims at giving its nurses a complete training not in its own interests but in theirs, and in that of the public. For this reason there is justice in the contention that the hospitals should receive grants in aid to cover the cost of their educational work.

It would be easy to disentangle the sums expended in training nurses over and above the needs of the institution. Modern requirements necessitate the appointment in all large schools of a tutor charged with the supervision of the probationers' entire instruction. Add to the sister-tutor's salary the cost of the teaching staff, the cost of special examiners, of class-rooms and apparatus, of special departments maintained for demonstration purposes. In large establishments the cost of these additional advantages runs to several hundred pounds a year. Even in the smallest it cannot be less than a hundred. To receive some portion, at any rate, of these charges back in the form of a grant would not only relieve the strain of finance, it would encourage a higher standard of education in many backward institutions, and make possible that levelling up of the entire profession at which the General Nursing Council is aiming. Supported as they are by the recommendation of the Cave Committee, the hospitals have a very strong case for assistance in the costly business of training nurses.

